# Supplementary material for: WordCommentsAnalyzer: A windows software tool for qualitative research
Source: F1000Res. 2018 Sep 4;7:536. Originally published 2018 May 3. [Version 2] doi: 10.12688/f1000research.14819.2 (PMC6137411; doi:10.12688/f1000research.14819.2)
Supplement: Supplementary file 3 [file f1000research-7-17614-s0002.tgz › b2a79496-4ada-47c4-9682-a99ce867836d.zip!/Eight Iranian Health Journals/Yafteh _46.docx]

© Yafteh  . This work is licensed under CC BY 4.0 (https://creativecommons.org/licenses/by/4.0/).

Volume & Issue: دوره 9 - شماره 3

Title: بررسی شیوع لپتوسپیروزیز در شالیکاران منطقه ویسیان و مقایسه آن با دامداران ازنا استان لرستان سال 1385

http://yafte.lums.ac.ir/article-1-41-fa.html

مقدمه: لپتوسپیروزیز یکی از بیماریهای عفونی اسپیروکتی و قابل انتقال بین حیوان و انسان میباشد. آب آلوده حامل باکتری است و لذا این بیماری در مناطق پرباران و در میان حرفه های در تماس با آب آلوده وحیوانات بیشتر مشاهده می شود. . علائم بالینی عفونت در 90 درصد موارد غیر اختصاصی و بشکل آنفلونزا بروز پیدا می کنند. لپتوسپیروزیز حاد میتواند چند عضو بدن از جمله کبد (سندرم ویلز)، کلیه، ریه و قلب را درگیر نماید. اثرات آن بخصوص بر جنین و در افراد مسن کشنده است. علیرغم انتشار جهانی آن متاسفانه در کشور ما عفونت لپتوسپیروزیز تشخیص داده نمی شود و لذا قبل از تشخیص اختصاصی با مصرف انواع آنتی بیوتیکها درمان میشود. ضمن انکه کیت تشخیص ان نیز در بیشتر آزمایشگاه ها فراهم نمی باشد. مواد و روشها: درشهریور و مهر 1384 از افرادی که با علائم آنفلونزا شامل تب و درد بدن به درمانگاه ویسیان مراجعه کردند و سابقه کار در شالیزار در آن سال داشتند 5 میلی لیتر خون وریدی گرفته و پرسشنامه پر شد. سپس سرمها از نظر وجود آنتی بادی Ig M و IgG باکیت آلمانی آزمایش شد. Virion/ Serion Classic IgG / IgM ELISA (Germany) برای از بین بردن فاکتور روماتو ئید IgM از کیت همراه Serion-Rhumatoid Factor . در آزمایش استفاده شد. یافته ها: آنتی بادی ضد لپتوسپیرا در خون 80 نفر از بیماران شالیکار ویسیان مورد آزمایش قرار گرفت. نتایج نشان داد که 8/48 درصد، شالیکاران آنتی بادی IgG و 75/ 38 درصد IgM ضد لپتوسپیرا دارند. بیشترین فراوانی در سن 41 سال به بالا دیده می شود. و از نظر جنسیت 92/76 درصد افراد مثبت منطقه ویسیان مرد و 08/23 درصد (9نفر) زن بودند. هیچ رابطه معنی داری بین نوع آب اشامیدنی مصرفی، سابقه سقط در دام یا خانواده بیماران بدست نیامد. مقایسه نتایج با دامداران غیر شالیکار و گزارشهای گیلان و بوشهر تاثیر آب و هوا وتماس شغلی را در میزان شیوع این عفونت را نشان داد. بحث و نتیجه گیری: لپتوسپیروزیز در سراسر ایران اتفاق می افتد اما تشخیص اختصاصی داده نمی شود و از آمار آن اطلاع دقیقی در دست نیست. افراد شاغل در شالیزارهای سراسر ایران دو برابر بیشتر در معرض خطر عفونت این اسیپروکت هستند و بایستی شرایط حفاظتی مناسب برای آنها فراهم گردد. ضمن آنکه امکانات سراسری تشخیص اختصاصی این میکروب برای انسان و دامها بایستی فراهم گردد.

Title: بررسی مولکولی آللهای DQB1،HLA-DRB1 در مبتلایان به کیست هیداتیک

http://yafte.lums.ac.ir/article-1-42-fa.html

مقدمه: هیداتیدوز یکی از بیماریهای انگلی مهم است که با استقرار مرحله لاروی کرم اکینوکوکوس گرانولوزوس سگ در میزبانان علفخوار واسط مثل گوسفندوگاوو...و هم چنین انسان، ایجاد می شود. تفاوت در حساسیت و مقاومت به ابتلا و عود در بیماریهای عفونی ازجمله کیست هیداتیک و آلوئولار در حیوانات وانسان دیده شده است که به دلیل تفاوت درفاکتورهای فردی و پاسخهای ایمنی، مبتنی بر تفاوت در ژنهای کد کننده آنتی ژنهای لوکوسیت های انسان و یا حیوان، می باشد. هدف از این تحقیق که برای اولین بار در ایران بر روی بیماران هیداتید انجام می شود، بررسی آللهای DRB1 و DRB1 ل وکوسیت انسان در منطقه است که در ایجاد مقاومت یا حساسیت به آلودگی کیست هیداتیک نقش دارند. مواد و روشها: برای شناسایی آللهای DQB1 (23 واکنش)، DRB1 (8 واکنش) با استفاده از ژنوم انسانی، 56 بیمارایرانی مبتلا به کیست هیداتیک تشخیص داده شده و یا جراحی شده و 30 فرد سالم با روش PCR-SSP (Polymerase Chain Reaction - Sequence Specific Primer) آزمایش شدند. DNA نمونه ها از خون کامل تخلیص گردید و PCR با استفاده از پرایمرهای اختصاصی انجام شد. پس از الکتروفورز محصول روی ژل آگارز نتایج با دستگاه UV خوانده وآللهای شناسایی شده, ثبت شد. آنالیز نتایج مولکولی ابتدا بااستفاده از نرم افزار INNO TRAIN و جداول فراوانی و تفسیر آللها برای تعیین ترکیب آللی( Allele Combination ) انجام و پس از آن آزمون همگنی با استفاده از2 χ و تست دقیق فیشرانجام شد. برای نمونه های معنی دار تحلیل رگرسیون لجستیک انجام و Odds - ratio آنها محاس به گردید. یافته ها : نتایج نشان داد که از بین ژنهای بررسی شده DQB1 ، DRB1 دربیماران و افراد سالم فراوانی آلل DQB1*03 با اختلاف معنی داری در گروه غیر هیداتیدی بالاتر است (P<0.02) و(87/2 Odds ratio = ).ومصونیت این افراد در صورت مواجهه با عامل بیماری در مقایسه با افراد فاقد این آلل حدود 87/2 برابر می باشد ودارندگان این آلل مقاومت بیشتری در برابر استقرار و رشد کیست هیداتیک تک حفره ای دارند. بحث و نتیجه گیری : این مطالعه اختلاف زمینه های ژنتیکی افراد در ابتلا به کیست هیداتیک را در منطقه مورد بررسی نشان داد و مشخص کرد که آلل DQB1*03 در ایجاد مقاومت بیشتر در برابر استقرار و رشد کیست هیداتیک تک حفره ای نقش دارد. مطالعات تکمیلی با روشهای مولکولی با قدرت تفکیک بالاتر پیشنهاد می گردد.

Title: مقایسه تاثیر درمانی نورتریپتیلین و سیتالوپرام در درمان اختلال افسردگی اساسی و مقایسه عوارض جانبی آنها

http://yafte.lums.ac.ir/article-1-43-fa.html

مقدمه: افسردگی اساسی یکی از شایعترین اختلالات روانی در تمام دنیا می باشد. درمان موثر این بیماری موجب حفظ عملکرد بیمار در سطح قابل قبول می گردد. داروهای مختلفی در درمان افسردگی اساسی به کار برده می شود. که هرکدام از این داروها تاثیر درمانی و عوارض جانبی خاص خود را دارد. از آنجا که داروهای SSRI اخیراً برای درمان این اختلال به کار برده می شوند و هنوز مطالعات زیادی در خصوص آنها به عمل نیامده است، بر آن شدیم مطالعه ای در خصوص مقایسه تاثیر درمانی نور تریپتیلین و سیتالوپرام در درمان اختلال افسردگی و مقایسه عوارض جانبی آنها انجام دهیم. مواد و روش‌ها: مطالعه حاضر یک مطالعه کارآزمایی بالینی دوسوکور می باشد. نمونه ها شامل 80 بیمار مبتلا به اختلال افسردگی اساسی (طبق معیارهای DSMIV T-R ) بوده که سابقه قبلی اختلال خلقی یا هر بیماری روانپزشکی شناخته شده قبلی یا سابقه بیماری های جسمی مزمن و سوءمصرف مواد نداشتند. نمونه ها به صورت تصادفی در دو گروه تحت درمان با سیتالوپرام و نورتریپتیلین قرار داده شدند. کلیه نمونه ها در هفته های 2، 4، 6، 8، 10 و 12 پس از شروع درمان از نظر عوارض دارویی و تاثیر درمانی مورد ارزیابی قرار گرفتند. اطلاعات پس از جمع آوری از طریق نرم افزار SPSS مورد تجزیه و تحلیل قرار گرفتند. یافته‌ها: نتایج نشانگر تاثیر داروهای سیتالوپرام و نورتریپتیلین در درمان افسردگی اساسی به یک اندازه بود. همچنین تفاوت معنی داری در میانگین نمرات وجود نداشت.در مقایسه عوارض دارویی نتایج نشان داد که بین بروز عارضه های پرخوابی، خشکی دهان، بی اشتهایی و تهوع تفاوت معنی دار بود. در حالی که بین بروز عوارض دیگر مثل اختلالات جنسی، کم خوابی، سردرد، لرزش ناشی از دارو، سرگیجه، چاقی، اسهال، درد شکم و استفراغ تفاوت معنی داری مشاهده نشد. بحث و نتیجه‌گیری: با توجه به یافته های فوق و نتایج مطالعات مشابه داروهای سه حلقه ای به اندازه داروهای SSRI در درمان افسردگی اساسی موثرند اما از نظر پروفایل عوارض جانبی با هم متفاوتند لذا با توجه به کم بودن داروها می توان نسبت به تجویز آنها اقدام نمود

Title: ارتباط هموسیستئین، ویتامین B12 و اسید فولیک سرم با بیماری حوادث عروق مغزی ایسکیمیک

http://yafte.lums.ac.ir/article-1-44-fa.html

مقدمه : سکته مغزی یکی از عوامل مهم مرگ و میر و معلولیت در جهان می باشد، عوامل خطر متعدد تغذیه ای و محیطی مانند دریافت ناکافی ویتامین B12 و اسید فولیک و بدنبال آن افزایش هموسیستئین تام سرم احتمال خطر بیماری عروقی مغز را افزایش می دهد. مواد و روشها: در این مطالعه مقطعی مورد - شاهدی تعداد 45 نفر از بیماران مبتلا به بیماری حوادث عروق مغزی ایسکیمیک و 75-45 ساله بستری در بخش و 45 نفر از افراد سالم نفر بودند که از نظر سن و جنس با یکدیگر جفت شده بودند، انتخاب و از هر کدام از نمونه ها مقدار 5 میلی لیتر خون وریدی جهت اندازه گیری سطح هموسیستئین، ویتامین B12 و اسید فولیک گرفته شد. میزان هموسیستئین سرم به روش الایزا و مقدار ویتامین B12 و اسید فولیک به روش رادیوایمونواسی اندازه گیری گردید. در این مطالعه برای مق ایسه اختلاف بین میانگین ها در دو گروه مستقل از آزمون استیودنت و همچنین برای تعیین ارتباط هموسیستئین با ویتامین B12 و اسید فولیک از مدل رگرسیون خطی استفاده شده است. یافته ها: براساس نتایج حاصل، میانگین سنی، قد و وزن در افراد شاهد به ترتیب 40/1 ± 95/60 ، 32/1± 41/163 و 02/2 ± 06/69 و در افراد بیمار به ترتیب 53/1 ± 39/61، 07/1 ± 86/163 و 62/1 ± 16/70 بود. از نظر BMI میانگین آن در افراد بیمار 51/0 ± kg / m2 30/26 و در افراد سالم 5/0 ± 68/25 بود. در گروه بیمار نسبت به گروه شاهد، سطح سرمی هموسیستئین بطور معنی داری بالاتر بود و سطح سرمی ویتامین B12 و نیز سطوح فولات سرم در گروه بیمار نسبت به گروه شاهد به طور معنی دار کمتر بود. بین هموسیستئین و فولات سرم و نیز هموسیستئین و ویتامین B12 یک ارتباط معنی دار منفی و معکوس وجود داشت بطوریکه با توجه به میزان R2 8%، تغییرات هموسیستئین با تغییرات اسید فولیک، همچنین با توجه به میزان R2 10%، تغییرات ویتامین B12 قابل توجیه است. بحث و نتیجه گیری : با توجه به نتایج حاصل از این مطالعه کمبود و دریافت ناکافی منابع غذایی ویتامین B12 و اسید فولیک موجب افزایش سطح سرمی هموسیستئین و عوارض ناشی از آن شده و نهایتاً بیماریهای عروقی و شانس ابتلا به حوادث عروقی مغزی ایسکیمیک را افزایش می دهد. لذا پیشنهاد می شود به منظور پیشگیری از افزایش هموسیستئین تام سرم از رژیم غذایی مناسب حاوی منابع اسید فولیک و ویتامین B12 استفاده شود

Title: میزان شیوع اختلالهای رفتار ایذائی در کودکان دبستانی شهر خرم آباد در سال 1384

http://yafte.lums.ac.ir/article-1-45-fa.html

مقدمه : اختلالات رفتار ایذائی از شایع‌ترین اختلالات روانپزشکی است که در کودکی آغاز می‌شود و یک معضل مهم بهداشت روانی جامعه به شمار می‌رود. اختلالات رفتار ایذائی اثرات مخربی بر روابط اجتماعی، تحصیلی، شخصیتی و رفتاری افراد در کودکی و بزرگسالی دارد. بنابر این بر آن شدیم پژوهش حاضر را با هدف بررسی شیوع اختلالات رفتار ایذائی در دانش‌آموزان دبستانی شهر خرم‌آباد انجام دهیم. مواد و روش‌ها: این پژوهش یک مطالعه Cross- Sectional است. جامعه آماری پژوهش شامل کلیه دانش‌آموزان مشغول به ‌تحصیل سال1384 در پایه‌های اول تا پنجم دبستان‌های شهر خرم‌آباد می‌باشد. (943 N= ). 16 مدرسه دولتی و غیردولتی (8 مدرسه دخـترانه، 8 مدرسه پسرانه) بصورت خوشه‌ای و چند مرحله‌ای انتخاب شدند. ابزار گردآوری اطلاعات پرسشـنامه استاندارد شده Child Symptom Inventories-4 (CSI-4) است که ابزاری است برای غربالگری شایع‌ترین اختلالهای روانپزشکی کودکان. پرسشنامه‌ها در دیداری جداگانه توسط والدین و معلمین تکمیل گردید. سپس نتایج توسط آمار توصیفی و آزمون مورد تجزیه و تحلیل قرار گرفت. یافته‌ها: از مجموع 943 نمونه، 4/21% دارای اختلال‌های رفتار ایذائی می‌باشند (7/17% اختلال‌ها بی‌اعتنایی مقابله‌ای و 7/3% اختلال سلوک). این میـزان در پسـران دو برابر دختـران بوده است (7/28% در مقابل 4/14%). بیشترین میزان اختلال رفتار ایذائی به ترتیب در پایه‌های سوم، چهـارم و پنجم و کمترین میزان در پایه دوم بود. همچنین بـین شیوع اختلالات رفتار ایذائی با جنس (P<0.001) ، پایه تحصیلی (P = 0.02) ، سواد والدین (P = 0.005 , P = 0.006) ، شغل مادر (P = 0.03) ، میزان درآمد ماهیانه (P=0.005) ارتباط معنی داری وجود داشت. ولی بین اختلالات رفتار ایذائی با شغل پدر، وضعیت تحصیلی دانش‌آموزان و مشکلات روانی خانواده ارتباط معنی‌داری وجود نداشت. بحث و نتیجه‌گیری: با توجه به شیوع بالای اختلالات رفتار ایذائی و عدم توجه کافی به عواقب ناشی از این اختلالات در گروه سنی کودک و نوجوان شناسائی این اختلالات ضروری به نظر می‌رسد تا مداخلات لازم و به موقع صورت گیرد.

Title: ارزیابی دو روش TRAP و FRAP جهت تعیین پتانسیل آنتی اکسیدانی تام سرم موش صحرایی

http://yafte.lums.ac.ir/article-1-46-fa.html

مقدمه : با توجه به اینکه مواد آنتی اکسیدان موجود در سرم یا پلاسما متعدد بوده و همچنین میانکنش های بین هر آنتی اکسیدان و شناساگر مربوطه متفاوت می باشد، لذا روشهای مختلفی برای تعیین پتانسیل آنتی اکسیدانی تام، بوجود آمده است. در این تحقیق، دو روش TRAP و FRAP جهت اندازه گیری پتانسیل آنتی اکسیدانی تام سرم موش، مورد ارزیابی و مقایسه قرار گرفت. مواد و روش‌ها: جهت تهیه نمونه خون، تعداد 10 موش صحرایی (جنس نر، نژاد ویستار) با میانگین وزنی 5 ± 190 گرم و سن 8 هفته به طور تصادفی انتخاب گردیدند. برای اندازه گیری پتانسیل آنتی اکسیدانی تام سرم موش از روش TRAP ، میزان کاهش جذب ماده فلورسانت R -فیکواریترین در حضور رادیکال AAPH و سرم خون بوسیله اسپکتروفلوریمتر اندازه گیری شد. در روش FRAP ، افزایش جذب کمپلکس -TPTZ کلریدآهن ( III ) توسط آنتی اکسیدان موجود در سرم در طول موج 593 نانومتر بوسیله اسپکتروفتومتر اندازه گیری گردید. نتایج بدست آمده از هر دو روش با استفاده از استاندارد Trolox به واحد m mol Trolox equivalent/L تبدیل گردید. یافته‌ها: نتایج این بررسی نشان داد که زمان نمونه گیری تاثیری بر میزان پتانسیل آنتی اکسیدانی تام سرم موش ها ندارد. همچنین مقایسه نتایج بدست آمده از دو روش TRAP و FRAP نشان داد که مقدار پتانسیل آنتی اکسیدانی تام بدست آمده از روش TRAP تقریبا 3 مرتبه از روش FRAP بالاتر است. بیشترین درصد پتانسیل آنتی اکسیدانی تام سرم بدست آمده از روش TRAP مربوط به آلبومین (33/30%) و در روش FRAP مربوط به آنتی اکسیدان اسید اوریک (13/57%) بود. علاوه بر این، میزان سهم آنتی اکسیدان β-کاروتن در روش TRAP برابر با 22/7% درحالیکه در روش FRAP برابر با صفر درصد بود. بحث و نتیجه گیری: پتانسیل آنتی اکسیدانی یک ماده آنتی اکسیدان برعلیه رادیکالهای آزاد، ضرورتا با توانایی آن ماده در احیاء آهن فریک به آهن فروس برابر نمی باشد

Title: میزان سم آفلاتوکسین M1 در شیرهای گاو محلی و پاستوریزه شهرستان خرم آباد به روش HPLC

http://yafte.lums.ac.ir/article-1-47-fa.html

مقدمه: آفلاتوکسین M1 فرم هیدروکسیله آفلاتوکسین B1 سمی است از قارچ آسپرژیلوس فلاووس که از طریق علوفه و نان کپک زده آلوده به این قارچ وارد بدن گاو یا سایر حیوانات علفخوار می شود و سپس در شیر ترشح می شود و دارای قدرت کارسینوژنیک قوی در کبد و کلیه می باشد. شیر که از پر مصرف ترین مواد غذایی در انسانها می باشد در معرض آلودگی زیادی به آن قرار دارد. مواد و روش‌ها: 42 نمونه از شیرهای محلی مراکز جمع آوری شیر و 40 نمونه از شیرهای پاستوریزه توزیع شده در شهرستان خرم آباد در سال 1384 در دو فصل تابستان و زمستان، از نظر سم آفلاتوکسین M1 ( AFM1 ) با روش HPLC و ستونهای ایمنوافینیتی بررسی شدند. یافته‌ها: 5 درصد نمونه های محلی در فصل تابستان با محدوده سمی ng/ml 046/0 – 017/0 و تمام نمونه های محلی فصل زمستان (100 درصد) با محدوده ng/ml 041/0-003/0 آلودگی به سم AFM1 را نشان دادند. همچنین در نمونه های پاستوریزه، در تابستان، 55 درصد با محدوده سمی ng/ml 533/0-017/0 و تمام نمونه های زمستان با محدوده ng/ml 054/0-005/0 آلودگی به سم AFM1 را نشان دادند. ازتمام نمونه ها در 2 فصل فقط یک نمونه با غلظت ng/ml 533/0 از پاستوریزه ها ی تابستان بیشتر از حد مجاز بود و بقیه نمونه ها کمتر از این حد را نشان دادند. میانگین آلودگی در نمونه های پاستوریزه و محلی در دو فصل تفاوت معنی دار نداشتند. بحث و نتیجه‌گیری: در نهایت نتایج حاصل از این پژوهش وجود آلودگی به سم AFM1 را در شیرهای پاستوریزه و محلی شهرستان خرم آباد را مطرح می کند هر چند که لزوم بررسی بیشترتوصیه می شود

Title: مقایسه ظرفیت تام آنتی اکسیدان سرمی و دریافت مواد غذایی اصلی حاوی آنتی اکسیدان در بیماران مبتلا به MS با افراد سالم

http://yafte.lums.ac.ir/article-1-48-fa.html

مقدمه : مولتیپل اسکلروز ( MS ) بیماری دمینیله شدن مزمن التهابی در سیستم عصبی مرکزی است که معمولا بین سنین 40-20 سالگی بروز پیدا می کند و در زنان نسبت به مردان شایع تر است. علت MS تاکنون ناشناحته باقی مانده و ژنتیک، عوامل عفونی و فرآیند های خود ایمنی که ممکن است تحت تاثیر عوامل محیطی شروع و ایجاد شوند ، از علل بیمارس هستند. هدف از مطالعه حاضر مقایسه وضعیت تام آنتی اکسیدانی ( TAS ) سرم و دریافت غذایی منابع عمده آنتی اکسیدانی در بیماران MS با نمونه های سالم بوده است مواد و روش‌ها: در این مطالعه که به روش مورد - شاهد ی انجام شده است، 21 بیمار مبتلا به MS (16 زن) با میانگین سنی 33 سال که توسط انجمن بیماران MS استان خوزستان در بیمارستان گلستان اهواز پذیرفته و علائم بیماری آنان توسط MRI تائید شده بود، شرکت داشتند. برای ارزیابی میزان دریافت منابع غذایی غنی از آنتی اکسیدان از کلیه افراد مورد مطالعه خواسته شد تا پرسشنامه نیمه کمی بسامد مواد غذایی را تکمیل کنند. تجزیه و تحلیل اطلاعات تغذیه ای توسط نرم افزار Nutritrack ویرایش 2004 انجام شد. نرم افزار آماری مورد استفاده SPSS ویرایش سیزدهم در نظر گرفته شد. یافته‌ها: در افراد مورد مطالعه بین وزن، قد و سن، BMI و همچنین سطح سرمی ظرفیت تام آنتی اکسیدان در افراد بیمار و گروه شاهد تفاوت معنی داری وجود نداشت، بین دریافت روزانه ویتامین های E, C , A و فولات بین دو گروه و بین مصرف گروه های غذایی عمده حاوی آنتی اکسیدان بین دو گروه تفاوت معنی داری مشاهده نشد. بحث و نتیجه‌گیری: با توجه به یافته های اندک فعلی، برای بیان رابطه آنتی اکسیدانهای رژیم غذایی با بروز و شدت بیماری MS به شواهد علمی بیشتری نیاز است و نتیجه گیری قطعی در حال حاضر امکان پذیر نیست.

Title: بررسی میزان انرژی درون سلولی در سلول های آپوپتوتیک

http://yafte.lums.ac.ir/article-1-49-fa.html

مقدمه: آپوپتوسیس فرآیندی فعال می باشد بطوری که فعالیت آنزیمها،تراکم کروماتین و تشکیل اجسام آپوپتوتیک نیازمند صرف انرژی است و لذا این احتمال وجود دارد که کاهش قابل توجهی درمیزان انرژی موجود در سلول های آپوپتوتیک صورت نگیرد.هدف از این تحقیق بررسی میزانATP در سلول های آپوپتوتیک می باشد. Ø موادمواد و روشها: این تحقیق یک مطالعه تجربی است که در آن از ٥٠ سر موش نژاد BALB/c در دو گروه استفاده شد. در گروه آزمون میزان mg/kg٢٠دگزامتازون ودر گروه شاهد نیز بافر سدیم فسفات بصورت داخل صفاقی تزریق صورت گرفت.١٦ ساعت پس از تزریق،غدد تیموس خارج گردید. یک لب هر غده جهت مطالعه میکروسکوپی ولب دیگر به منظور بررسی میزان ATP درون سلولی با استفاده از تکنیک رزونانس مغناطیس هسته مورد استفاده قرار گرفت. یافته ها: بر اساس نتایج حاصل از این تحقیق،میزان ATP در سلول های آپوپتوتیک حدود٨٦ % سلو ل های سالم بوده است بحث و نتیجه گیری: این مطالعه بیانگر فعال بودن روند مرگ سلولی آپوپتوسیس و نیز عدم کاهش شدید میزانATP این سلول ها در طی مرگ سلولی می باشد

Title: تأثیر گیاهان میخک، سیاه دانه و بید مجنون بر زگیل و مقایسه ی آن با درمان های معمول

http://yafte.lums.ac.ir/article-1-50-fa.html

مقدمه : زگیل یک بیماری ویروسی پوستی شایع و قابل انتقال است که به وسیله ی پاپیلوما ویروس ها ایجاد و منجر به مشکلات زیبائی، روانی و در صورت بروز در نواحی تحت فشار و تماس، سبب درد و التهاب می گردد. درمان های دارویی و غیر دارویی نیز برای زگیل انجام می شود، مثل تجویز پودوفیلین که یک رزین با منشأ گیاهی حاوی ترکیبات سیتوتوکسیک است و در زگیل ژنیتال استفاده می شود. در این مطالعه، تأثیر گیاهان سیاه دانه، میخک و بید مجنون با روغن زیتون در درمان زگیل و مقایسه آن با درمان معمول مورد بررسی قرار گرفته است مواد و روشها: این بررسی، یک کارآزمایی بالینی است که افراد مورد مطالعه، دانش آموزان دختر مقطع راهنمایی و دبیرستان شهر خرم آباد در سال 1386 بودند. نمونه ها با روش تصادفی ( رندوم- کنترل ) باتوجه به وجود معیارهای ورود به مطالعه انتخاب و به سه گروه 97 نفره تقسیم شدند. در پایان، همه پاسخ ها تنظیم و وارد برنامه SPSS نسخه ی 14 شده و مورد تجزیه و تحلیل آماری قرار گرفتند. یافته‌ها: تعداد کل واحدهای مورد مطالعه در مرحله ی اول بررسی، 291 نفر بودند. سن آن ها در محدوده ی 12 تا 18 سال بود. 41/64 % ضایعات بر روی دست ها و بقیه در سایر نواحی بود. 4% افراد به درمان معمول و 1% به درمان با داروهای گیاهی با اسید حساسیت موضعی نشان دادند. از کل بیماران، 177 نفر درمان ها را به صورت مرتب استفاده کرده بودند ( 61 نفر درمان معمول، 52 نفر درمان گیاهی با اسید و 64 نفر درمان گیاهی بدون اسید ). بحث و نتیجه‌گیری: استفاده از داروی گیاهی با و بدون اسید، تأثیر قابل ملاحظه ای بر درمان ضایعات داشت. البته، تفاوت تأثیر درمان معمول نسبت به داروی گیاهی بدون اسید بسیار کم و ازنظر آماری معنی دار نبود. در حالی که، تأثیر درمان معمول نسبت به داروی گیاهی با اسید بیشتر بود. باتوجه به نتایج این مطالعه، می توان از گیاهان فوق به عنوان درمان مکمل یا جایگزین در مبتلایان به زگیل معمولی استفاده نمود.

Title: ارزیابی اثر فوتوالکترون تراپی بر روی بیماران مبتلا به هپاتوسلولار کارسینوما

http://yafte.lums.ac.ir/article-1-51-fa.html

مقدمه : روش جدید فوتو الکترون تراپی قبلاً بر روی فانتوم، محیط های کشت سلولهای سرطانی و حیوانات آزمایشگاهی با موفقیت اجرا گردیده است. در این روش ابتدا داروهای حاوی مواد حاجب پرتو ایکس که عدد اتمی بالایی دارند به بدن بیماران تزریق می شود و پس از تجمع حداقل ده درصد از دارو در محل تومور در یک جلسه تابش پرتو ایکس 200 تا 300 کیلوولت بصورت لوکال به ناحیه تومور انجام می شود. کمیته اخلاق پزشکی اولین گروه بیماران کاندید درمان با روش فوتوالکترون تراپی را هپاتوسلولار کارسینوما تعیین نمود. هپاتوسلولار کارسینوما یکی از بدخیمی های شایع ناحیه کبد می باشد. طولانی کردن عمر هدف اصلی برای بیشتر این بیماران است و در اغلب موارد این هدف با روشهای غیر جراحی قابل وصول است. این روشها شامل شیمی درمانی، رادیوتراپی، استفاده از امواج RF ( Radio Frequency )، ( TACE ) Trans Artepical Chemo Embolization و ( PEI ) Percutaneous Ethanol Injection هستند. میزان پاسخ این روشها به تنهایی و به صورت توأم 10% تا 47% گزارش شده است . مواد و روش‌ها: این تحقیق به صورت ‏کارآزمایی بالینی‏ اجرا گردید. در این روش ابتدا داروی ‏لیپیدول‏ که یک ماده حاجب پرتو ایکس با عدد اتمی بالا است از طریق آنژیو کاتتر به شریان مشروب کننده تومور منتقل شده، سپس در یک جلسه پرتو ایکس 200 تا 250 کیلوالکترون ولت بصورت ‏موضعی به محل تومور تابیده می شود. دوز دارو متناسب با حجم تومور بین 20 تا 60 سانتیمتر مکعب و مقدار پرتو بین 400 تا 600 سانتی گری ‏بوده است. مقدار جذب پرتو در این دارو نسبت به بافت سالم بدن دهها برابر بیشتر است در نتیجه موجب جذب انرژی پرتو در ناحیه تومور و ‏نهایتاً آسیب خیلی شدید به این ناحیه می شود. یافته‌ها: تصاویر سی تی اسکن شش ماه پس از درمان یک جلسه ای هر شش بیماری که فوتوالکترون تراپی شدند نشان دهنده آسیب قابل توجه و اختصاصی به سلولهای تومور بودند. بحث و نتیجه گیری: اندزه گیریها کاهش اندازه تومور بین 40 تا 80 درصد را نشان می دادند که بطور قطع ناشی از آسیب کشنده به سلولهای تومور بوده است. با توجه باینکه بیماران این تحقیق از سیروز و هپاتیت B نیز رنج می بردند امکان استفاده از تست های کبدی برای ارزیابی وجود نداشت ولی تا همین حد نشان دهنده آسیب رساندن به ناحیه تومور بصورت هوشمند و لوکالیزه بوده اند.
